# Spatial Pattern Enhances Ecosystem Functioning in an African Savanna

**DOI:** 10.1371/journal.pbio.1000377

**Published:** 2010-05-25

**Authors:** Robert M. Pringle, Daniel F. Doak, Alison K. Brody, Rudy Jocqué, Todd M. Palmer

**Affiliations:** 1 Society of Fellows, Harvard University, Cambridge, Massachusetts, United States of America; 2 Mpala Research Centre, Nanyuki, Kenya; 3 Department of Zoology and Physiology, University of Wyoming, Laramie, Wyoming, United States of America; 4 Department of Biology, University of Vermont, Burlington, Vermont, United States of America; 5 Department of African Zoology, Royal Museum for Central Africa, Tervuren, Belgium; 6 Department of Biology, University of Florida, Gainesville, Florida, United States of America; McGill University, Canada

## Abstract

The finding that regular spatial patterns can emerge in nature from local interactions between organisms has prompted a search for the ecological importance of these patterns. Theoretical models have predicted that patterning may have positive emergent effects on fundamental ecosystem functions, such as productivity. We provide empirical support for this prediction. In dryland ecosystems, termite mounds are often hotspots of plant growth (primary productivity). Using detailed observations and manipulative experiments in an African savanna, we show that these mounds are also local hotspots of animal abundance (secondary and tertiary productivity): insect abundance and biomass decreased with distance from the nearest termite mound, as did the abundance, biomass, and reproductive output of insect-eating predators. Null-model analyses indicated that at the landscape scale, the evenly spaced distribution of termite mounds produced dramatically greater abundance, biomass, and reproductive output of consumers across trophic levels than would be obtained in landscapes with randomly distributed mounds. These emergent properties of spatial pattern arose because the average distance from an arbitrarily chosen point to the nearest feature in a landscape is minimized in landscapes where the features are hyper-dispersed (i.e., uniformly spaced). This suggests that the linkage between patterning and ecosystem functioning will be common to systems spanning the range of human management intensities. The centrality of spatial pattern to system-wide biomass accumulation underscores the need to conserve pattern-generating organisms and mechanisms, and to incorporate landscape patterning in efforts to restore degraded habitats and maximize the delivery of ecosystem services.

## Abstract 2

Termites indirectly enhance plant and animal productivity near their mounds, and the uniform spatial patterning of these mounds enhances the overall productivity of the entire landscape.

## Author Summary

Local interactions between organisms in nature can scale up to produce strikingly regular patterns across entire landscapes. With improvements in satellite imagery, such patterns are increasingly reported in the ecological literature. It remains unclear, however, whether the existence of such patterns actually matters for key ecosystem processes such as productivity. In semi-arid East Africa, below-ground mounds built by *Odontotermes* termites frequently occur in uniform, “polka-dot” arrangements. We show that, due to the ways in which termites modify the soil, these mounds are hotspots of plant and animal productivity: close to termite mounds, plants grow more quickly, herbivorous and predatory animals are more abundant, and reproductive output is greater than is true farther away from mounds. Moreover, the evenly spaced distribution of termite mounds means that all points in the landscape are relatively close to the nearest mound—with the result that ecosystem-wide productivity is greater under the actual distribution of mounds than it would be if the same number of mounds were randomly situated. Thus, although subterranean termites may be less visible and charismatic than the large mammals of African savannas, they are nonetheless critically important engineers of structures and patterns that underpin ecosystem function.

## Introduction

A succession of spatially explicit ecological models in the early 1990s indicated that large-scale regular spatial patterns could arise within homogeneous landscapes from local biotic interactions alone [1]–[3], with potentially profound implications for the maintenance of biodiversity and ecological stability [4],[5]. At first, large-scale ordered patterns were harder to find in natural systems than in systems of equations: the title of a 1997 review questioned whether ecological self-organization was “robust reality” or merely a theoretical set of “pretty patterns” [6].

Over the past decade, however, multiple studies have shown that regular patterns are both common and persistent across a range of ecosystems [7]–[10]. But the crucial questions of whether and how these patterns influence ecosystem functioning remain unanswered [11]. Here, we show that the even spacing of subterranean termite mounds in an apparently homogeneous African savanna provides a template for parallel spatial patterning in tree-dwelling animal communities. We further show that the uniformity of this pattern at small spatial scales elevates the productivity of the entire landscape, providing support for models linking spatial pattern with ecosystem functioning [12]–[15].

Our study site in central Kenya (0°20′ N, 36°53′ E) is a wooded grassland on level vertisol soils. The high clay concentration (40%–60%) of these soils reduces water infiltration and causes shrink-swell dynamics that can shear plant roots [16]. In this habitat, which is widespread in East Africa, a single *Acacia* species (*A. drepanolobium*, an “ant plant”) constitutes >97% of the canopy over a continuous understory dominated by five perennial bunchgrasses. Thus, the area appears strikingly homogeneous for a tropical terrestrial ecosystem (Figure S1). In addition to symbiotic ants (3 *Crematogaster* spp., 1 *Tetraponera* sp.), *A. drepanolobium* canopies are inhabited by non-predatory insects, predatory insects and spiders, and insect-eating dwarf geckos (*Lygodactylus keniensis*). *Lygodactylus keniensis* is diurnal and exclusively arboreal, and males are territorial; along with the arthropod arboreal predators in the system, it preys almost exclusively on tree-feeding insects [17], excepting workers of the *Acacia*-ant species [18].

**Figure 1:**
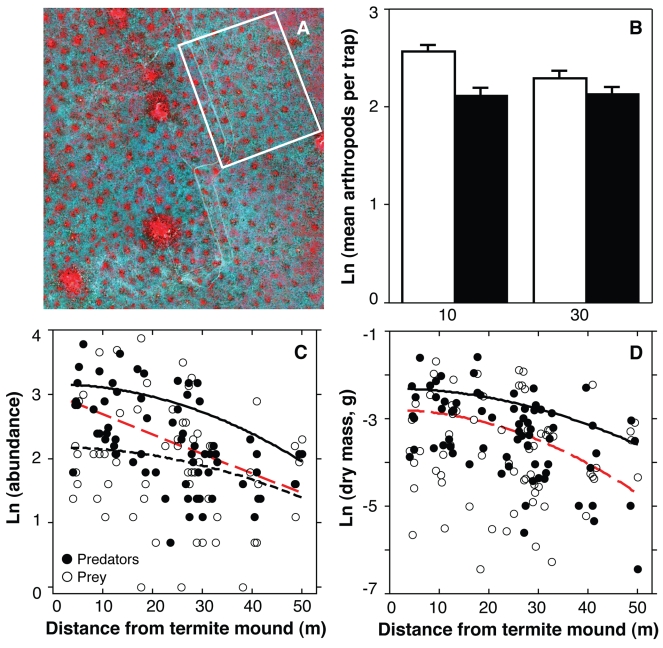
Patterns in arthropod communities. (A) Multispectral Quickbird satellite image (2.4 m resolution, here in false-color infrared) showing even spacing of termite mounds (small circular regions with red color indicative of high primary productivity; large red regions are abandoned cattle corrals). White rectangle encompasses the 0.36 km^2^ area mapped for analyses (see Figures 2B and 4). (B) Aerial-arthropod abundance. White bars represent sides of traps facing mounds (± SE), black bars the opposing sides (repeated-measures MANOVA: mound proximity *F*_1,33_ = 9.5, *p* = 0.004; orientation *F*_1,33_ = 49.0, *p*<0.0001; proximity × orientation *F*_1,33_ = 10.9, *p* = 0.002). (C) Arboreal-arthropod abundance. Fitted curves are regressions against raw mound proximity (for predators only; red-dashed line) and square-transformed mound proximity (for prey only and for all arthropods combined; black-dotted and solid lines, respectively). The form of these curves and the following tests of significance for mound-proximity were determined from the best-fitting multiple regressions in Table S3 (all arthropods combined: *F*_1,61_ = 22.6, *p*<0.0001; prey only: *F*_1,62_ = 4.9, *p* = 0.03; predators only: *F*_1,62_ = 49.3, *p*<0.0001). Omitting the 34 mantids, such that predators are represented by spiders only, does not change the results (*r* = −0.81, *F*_1,62_ = 42.8, *p*<0.0001). (D) Arboreal-arthropod biomasses as functions of mound-proximity squared. Statistics and symbols as above (all arthropods: *F*_1,62_ = 15.0, *p* = 0.0003; prey only [non-significant, no curve]: *F*_1,62_ = 0.7, *p* = 0.4; predators only: *F*_1,62_ = 26.5, *p*<0.0001; spiders only: *r* = −0.55, *F*_1,61_ = 16.1, *p* = 0.0002).

In this ecosystem, fungus-cultivating termites (Macrotermitinae: *Odontotermes*) nest within low, subterranean mounds (10–20 m diameter, <0.5 m tall) (Figure S1). As in many other drylands worldwide, these mounds occur in regular, over-dispersed (evenly spaced) spatial patterns (see Figure 1A) [19]–[21]. These patterned arrays are apparently maintained in space by termite colonies' competitive partitioning of the habitat into non-overlapping foraging territories [16],[21],[22] and in time by cycles of colony extinction and mound re-colonization (Text S1) [16],[21]. The mounds themselves are typically treeless and dominated by the perennial bunchgrass *Pennisetum stramineum* [23], and they are often centuries old [16],[21]. Mound surfaces contain more sand than the surrounding soils, which, along with termite-created macrochannels, increases aeration and water infiltration [24], decreases shrink-swell dynamics, and accelerates soil formation from bedrock [16]. Termite mounds are comparatively moist microenvironments in dry savannas [25], and mound soils are enriched in nitrogen and phosphorus (by 70% and 84%, respectively, relative to off-mound soils: [26]). This combination of physical and chemical properties results in greater production of grasses on termite mounds, which is clearly visible in multispectral satellite photographs using the near-infrared band (Figure 1A). Woody productivity is also enhanced on mound edges: *A. drepanolobium* foliar nitrogen content is 19% greater within 5 m from edges [26], new-shoot growth of trees is 60% greater within 10 m of edges [23], and trees adjacent to mounds are ∼120% more likely to fruit in a given season [26]. *Acacia* trees in nitrogen-rich near-mound soils rely less heavily on fixed atmospheric N than trees in the inter-mound matrix [27], providing one possible explanation for these patterns.

We used field observations and manipulative experiments to show that, by enhancing primary productivity on and around their mounds, termites exert positive indirect effects upon multiple trophic levels of arboreal animals, from herbivorous insects to spiders and geckos. We further show that these indirect effects create spatial patterns in the abundance and reproductive output of these taxa that parallel the patterning of termite mounds. We then extrapolated these patterns to the landscape scale, showing that uniform spacing of termite mounds (over-dispersion) increases secondary and tertiary productivity relative to simulated landscapes in which mounds were randomly distributed.

## Results/Discussion

We quantified the spatial pattern of termite mounds using Ripley's *K* [28], showing that they exhibit significant over-dispersion at spatial scales<100 m (Figure S2). We then quantified consumer abundances at different distances from mounds to determine whether this pattern of high-productivity patches provides a template for the distribution of prey and predator communities. Aerial arthropods (*N* = 3,277; 42% Hemiptera, 32% Diptera, 11% Coleoptera, 15% others) were significantly more abundant in sticky traps at 10 m than at 30 m from termite mounds (Figure 1B). Moreover, the sides of the traps facing the mounds captured nearly 40% more arthropods than the away-facing sides, and this discrepancy was more pronounced close to mounds. These results suggest that mounds are a local source of insects dispersing into the inter-mound matrix. For tree-dwelling arthropods, sampled by spraying with insecticide (*N* = 1,503; 55% spiders, 23% Coleoptera, 6% Lepidoptera), the abundance and biomass of all arthropods and of predatory taxa only, and the abundance (but not biomass) of prey taxa, decreased significantly with distance from mound centers (Figure 1C–D). More than 96% (824 of 858) predatory arthropods in our samples were spiders, so our conclusions about predatory arthropods in general are also true for spiders in particular. Only two of 4,780 (0.04%) total arthropods were termites (both alates captured in sticky traps), indicating that termites themselves were not driving the pattern in prey abundance or providing a prey base for arboreal insectivores (Text S2).

To determine whether the gecko *L. keniensis* was more abundant near mounds, we exhaustively searched 60 randomly selected trees in hemispheres around each of three mounds where all trees had been mapped (*N* = 180 trees total). On average, trees occupied by one or more geckos (*N* = 72) were significantly closer to mounds (median = 18.3, interquartile range = 12.6–26.3) than were unoccupied trees (*N* = 108, median = 26.4, interquartile range = 15.4–31.4; Wilcoxon *Z* = −3.6, *p* = 0.0003). We constructed a candidate set of 108 ordinal-logistic regression models to identify the factors influencing the number of geckos on trees. Ranking these models using the sample-size-corrected Akaike Information Criterion (AIC*_c_*) [29] revealed that the number of geckos on a tree was principally a function of the tree's size (estimated surface area of the main stem) and its proximity to the nearest termite mound (Table S1). The best model achieved good correspondence between observed and predicted values (Figure S3A) and showed strong predictive power when applied to a larger dataset (*N* = 477 trees) collected 3 y after the model was parameterized (August 2009; Figure S3B).

**Figure 2:**
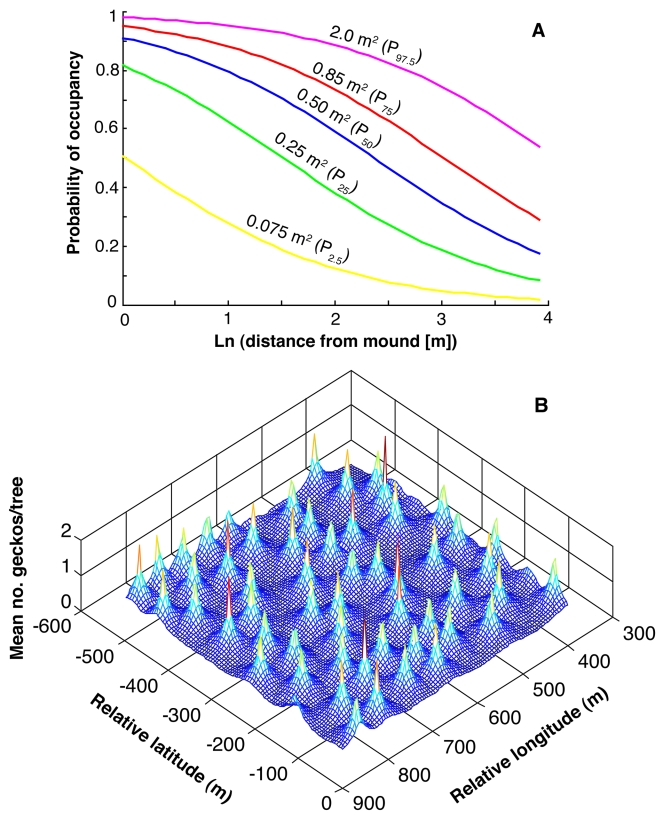
Gecko responses to termite mounds. (A) Probability of gecko occupancy (≥1 individual) as a function of mound proximity and five percentiles of observed tree surface area. (B) Spatial probability distribution of number of geckos per tree in a 0.36 km^2^ portion of the study site, assuming trees with median surface area. Expected values for each grid cell are drawn from the best-fitting ordinal-regression model of number of geckos per tree (Table S1).

Using the parameters of this model, we determined the mean probability of occupancy (≥ 1 gecko) as a function of mound proximity for five percentiles of tree size (Figure 2A), showing that whereas very large trees are nearly always occupied, occupancy of intermediate-sized trees hinges strongly on location relative to termite mounds. We then estimated the mean number of geckos expected on a tree of median size occurring anywhere in an actual landscape of mapped mounds within our study area, which revealed a strikingly uniform pattern in the spatial probability distribution of these predators (Figure 2B).

Two questions remain about the mechanisms causing this pattern. First, mean tree size decreased with distance from the nearest mound (*F*_1,475_ = 19.3, *p*<0.0001), suggesting non-independence of these two effects in the regression models. Second, it is not clear what drives the “mound-proximity” effect in the gecko regressions. The decrease in arthropod abundance with increasing distance from mounds suggests—but does not demonstrate—that geckos might be responding to differences in prey availability.

**Figure 3:**
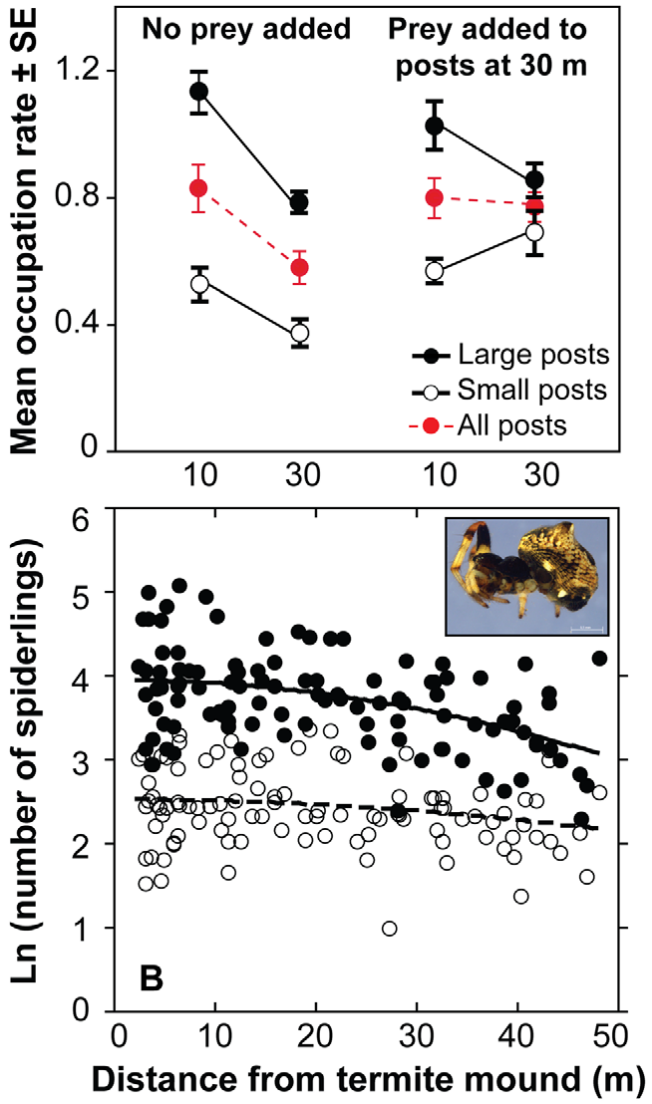
Termites' effects on arboreal predators. (A) Gecko habitat-selection experiment (repeated-measures MANOVA main effects: post size *F*_1,33_ = 95.9, *p*<0.0001; mound proximity *F*_1,33_ = 11.0, *p* = 0.002). Experimental prey supplementation at 30 m essentially equalized mean occupation rates at 10 m and 30 m (red markers; time × proximity interaction: *F*_1,33_ = 7.5, *p*<0.01; Table S3). (B) Fecundity of female *Cyclosa* sp. (inset) decreases as a function of mound-proximity squared. Statistical significance of mound proximity determined using multiple regression with female carapace width as a covariate (*N* = 102). For total offspring per female (solid circles): *F*_1,99_ = 21.9, *p*<0.0001. For mean number of offspring per egg sac per female (open circles): *F*_1,88_ = 5.1, *p* = 0.026.

We addressed these issues experimentally using artificial “trees” consisting of wooden posts of two sizes (“large” and “small”). These posts differed only in their size. At each of 12 mounds, we placed one post of each size at both 10 m (“close”) and 30 m (“far”) from the mound center, controlling for nearby tree density. From October 2006 to June 2007, we surveyed all posts 12 times. Occupation frequency was greater on large and close posts than small and far ones (Figure 3A), as our model predicted. Moreover, the mean snout-vent length and weight of territorial male geckos (but not females) was greater for close posts (but did not vary by post size) (univariate effect test of mound proximity from two-way ANOVA: *F*_1,32_ = 7.8, *p* = 0.009 for length; *F*_1,32_ = 4.5, *p* = 0.04 for weight).

Thus, tree size and mound proximity have independent effects on gecko occurrence. To test whether the effect of mound proximity indeed arose from variation in prey availability, we repeated the artificial-tree experiment with one modification: we affixed plastic cups to the base of every post and, each morning from August–December 2007, we added 3–9 non-flying insects to cups at all far posts only. Consistent with the prey-availability hypothesis, prey supplementation almost equalized the overall mean occupation frequencies at 10 m (0.799±0.064 geckos/post) and 30 m (0.771±0.046 geckos/post) from termite mounds (Figure 3A, Table S4, Text S3). We conclude that termites indirectly influence gecko distribution by increasing local densities of arthropod prey near mounds. That trees near mounds are on average larger, exhibit greater foliar N content [26] and growth rates [23], and rely more heavily on soil N than atmospheric N [27] all strongly suggest an additional likely mechanism: termite activity increases mean tree size and thus, indirectly, occupancy of those trees.

That adding prey increased occupation of far posts is consistent with a behavioral response to food availability but does not rule out a simultaneous numerical (i.e., reproductive) response. It is difficult to measure variation in reproductive output for *L. keniensis*, which has a fixed clutch size. But we can easily measure fecundity for another group of arboreal predators, female spiders, which produce conspicuous egg masses of variable size. (Multiple-regression and AIC*_c_* analyses of occupancy patterns showed that the abundance of adult spiders, like that of geckos, rapidly decreased with distance from the nearest termite mound; Table S2.) We haphazardly collected 110 reproductive females of the most common arboreal web spider (Araneidae: *Cyclosa* sp.) from April–June 2008 at varying distances from 12 non-adjacent mounds (∼9 spiders per mound) and reared their egg masses. Both the total number of spiderlings per female and the mean number of spiderlings per egg sac within each egg mass decreased with distance from mounds (Figure 3B), indicating that spiders respond numerically to high-productivity mound areas.

These results are a unique demonstration that subterranean termites indirectly enhance abundance and create spatial pattern across multiple trophic levels of tree-dwelling animals. We next tested the theoretical prediction [12]–[14] that the regularity of the spatial pattern should increase overall production at the landscape scale. We cannot easily conduct this test in the field: we lack “control” regions without patterned mounds, and the experimental elimination of termite colonies would not eliminate the gradient in production because the mound structures would likely persist for decades. We therefore employed a null-model approach. We first superimposed a grid of 5×5 m cells upon a ∼360,000 m^2^ mapped portion of the study site (see Figure 1A). We then used best-fitting regression models (selected from candidate sets using AIC*_c_*, as for geckos above; Tables S1–S3) to estimate the value of the response variables at each of the 14,400 sample points defined by this grid. (To isolate the effects of mound proximity, we set all other predictor variables equal to their observed median values.) Next, for each variable, we averaged the values of the 14,400 sample points to yield a landscape-mean value. (We refer to these values as “over-dispersed-landscape means” because each is based on the evenly spaced distribution of mapped mounds in the real landscape.) Finally, we compared the over-dispersed-landscape mean for each variable with “random-landscape means” obtained from applying the same models to 1,000 simulated landscapes of randomly distributed mounds (see Materials and Methods: spatial analysis of patterns in consumer abundance).

**Figure 4:**
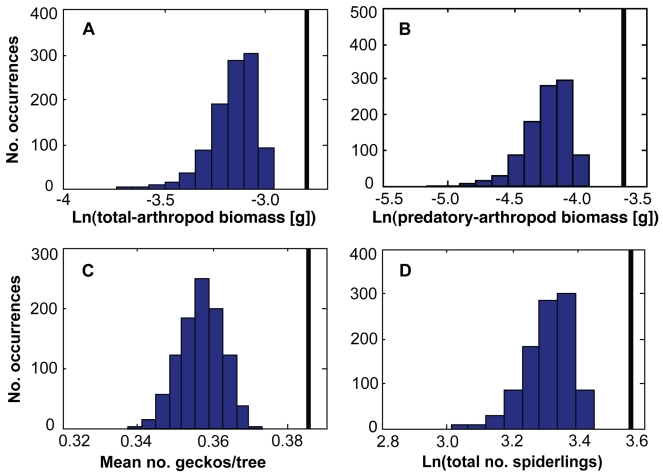
Frequency distributions of mean landscape values in 1,000 simulated landscapes of randomly placed mounds for (A) total-arthropod biomass, (B) predatory-arthropod biomass, (C) geckos, and (D) spider fecundity. Vertical bars show the mean landscape value for each variable obtained using the evenly spaced distribution of termite mounds in the mapped 0.36 km^2^ area of the landscape (Figure 1A). The best-fitting models used in these analyses are presented in Tables S1–S3. Results for total- and predatory-arthropod abundance are not shown but parallel those for biomass.

For every variable, the real (over-dispersed) landscape was far more productive than were simulated landscapes with randomly distributed mounds. The estimated means for all response variables in the over-dispersed landscape were >99.9th percentile of the means obtained from the 1,000 randomly generated landscapes (Figure 4). Because the mean values of all variables were strongly negatively correlated with mean nearest-mound distance (Figure S4), the uniform spacing of mounds in the real landscape—which minimizes the mean distance from any arbitrarily chosen point in the landscape to the nearest mound—maximizes mean values of the response variables.

This analysis assumes that multiple mounds would not have additive effects on the density or productivity of trees and tree fauna, which might lead to greater production under clumped scenarios than our models (which were based only on nearest-mound distance) predict. We tested this assumption. When we collected data on gecko abundances in 2009 to test the predictive power of our best-fitting ordinal regression model, we recorded the locations of the two mounds closest to each tree (hereafter, nearest and second-nearest). Adding second-nearest mound distance as a predictor to our best model did not improve the model whatsoever (−2×log-likelihood = 666.265641 for both models). Because nearest and second-nearest mound distances were very weakly correlated (*r* = −0.08), this result is not biased by collinearity of the predictors. We therefore conclude that the “mound effect” on production is adequately characterized by distance to the single nearest mound. Our simulation results also assume that trees are equally likely to occur anywhere in the landscape, so gradients in tree density might complicate our conclusions. In fact, the response of tree density to mound proximity is weak and inconsistent, and a separate set of landscape simulations in which we accounted for these effects (see Materials and Methods) produced qualitatively identical results (Figure S5).

Collectively, our data show (a) that a regularly patterned array of termite mounds induces parallel patterning in the abundance and reproductive output of tree-dwelling fauna, (b) that these patterns arise via both consumptive (i.e., trophic) and non-consumptive (i.e., engineering) indirect interactions, and (c) that the uniformity of the pattern increases the total biomass of prey and predators in the landscape. This emergent effect of spatial pattern upon a fundamental ecosystem function (productivity across trophic levels) confirms theory predicting linkages between patterning and production. Our results further imply that the landscape-level effects of any set of features that induce local gradients in ecological processes are likely to hinge on the spatial patterning of these features, with highly uniform spacing often producing the strongest net outcomes. Future work should address how the landscape-level effects of different spatial patterns vary with the shape and slope of biotic distance-response functions, as well as with possible interactive effects among patterned features.

Our study highlights the importance of conserving pattern-inducing taxa and processes—in this case, termites and their mound-building activities. In Africa's fields and pastures, termites are sometimes eradicated to protect crops and forage, and mounds are sometimes destroyed to redistribute the nutrients within them [20], yet these actions may actually diminish overall landscape productivity. More generally, recent research shows that the influence of remnant trees in forest regeneration attenuates with distance [30], which means that restoration efforts will be most effective if organisms—such as trees and corals intended as nucleating agents for forests and reefs—are added to landscapes in uniform, gridded patterns (as theory suggests: [14]). Conversely, other desired ecological outcomes, such as the persistence of competitively inferior plant species, may be most effective if elements are arranged in aggregated distributions [31]. The uniform spacing of plants in plantations, and the ability to manipulate the spatial configuration of the agroscape, likewise provides opportunities to both study and apply the consequences of spatial patterning for the delivery of ecosystem services such as pest control and pollination [32],[33].

## Materials and Methods

### Site Description

We conducted this study between June 2006 and August 2009 at the Mpala Research Centre (0°20′ N, 36°53′ E) in central Kenya. Total rainfall during this period was 1,810 mm. The annual pattern was variable and tri-modal, with peaks in August (70 mm) and November (93 mm) of 2006; April (86 mm), June (152 mm), and September (98 mm) of 2007; and May (99.6 mm), July (58.7 mm), and October (143.8 mm) of 2008, followed by drought. The study area is underlain by flat, heavy-clay vertisol (“black cotton”) soils of recent volcanic origin, which are characterized by impeded drainage, pronounced shrink-swell dynamics [34],[35], and species-poor plant communities [36]. These soils and associated vegetation occur in many parts of East Africa, including Nairobi National Park and the western extension of Serengeti National Park [37].

Each *A. drepanolobium* tree is inhabited by one of four species of symbiotic ants (*Crematogaster* and *Tetraponera* spp. [38]). Trees inhabited by each ant species support robust communities of insects, predatory arthropods (primarily spiders and mantids), and dwarf geckos (*Lygodactylus keniensis*). Because worker ants do not appear to be frequent prey for any of the arboreal predators we studied, we did not include them in our samples or surveys. Adult male *L. keniensis* are distinguished by a chevron-shaped row of pre-anal pores and fiercely defend territories consisting of individual trees or adjacent trees with contiguous canopies, while several females and subadults can occur on the same tree [18].

### Termite Mounds and Spatial Pattern

Nests built by subterranean termites (Macrotermitinae: *Odontotermes*) occur in this and similar habitats throughout upland East Africa. As described above, various physical, chemical, and hydrological properties of mounds lead to greater productivity of both woody and herbaceous plants, revealed at our sites by both field measurements [23],[26] and remotely sensed imagery (Figure 1A; see also [39]). Similar effects of termite mounds on primary productivity occur in many systems [20]. Like all Macrotermitinae, *Odontotermes* spp. farm fungus in combs underground. Alates typically emerge with the first heavy rain of the wet season [21], but workers and soldiers are virtually never exposed aboveground (see Results), foraging instead within covered runways on the soil surface.

Macrotermitinae mounds have long been known to occur with apparently even spacing in upland Kenya and other semi-arid regions throughout Africa (Figure 1A; [16],[19],[21]). Such regular spacing (20–120 m between mounds) arises from colonies' exhaustive partitioning of space into non-overlapping foraging areas (Text S1) [20]–[22]. We quantitatively evaluated mound patterning at different spatial scales using Ripley's *K* function [28]. Using the near-infrared band from an orthorectified Quickbird satellite image (June 20, 2003) with 2.4 m resolution and ∼3 km^2^ extent, we visually identified circular areas of high productivity, corresponding to termite mounds. To verify accuracy of our visual photo-interpretation, we field-recorded the geographic coordinates of 50 mounds using a CMT March II GPS (1–5 m accuracy), which we overlaid as a shape file upon the satellite image, confirming that these ground-truthed points did indeed appear as mounds on the image. We then applied Ripley's *K* to the coordinates of these mounds using Programita [40], establishing that the spacing of mounds is significantly uniform at spatial scales<100 m (Figure S2).

### Arthropod Surveys

We identified all arthropods to order and some spiders and beetles to family. For tree-dwelling arthropods, we analyzed predators and prey both separately and together. Because the ecology and taxonomy of the invertebrate fauna of this region is poorly characterized, we treated mantids and spiders as predators and assumed that all other insects represented “prey.” Although this categorization slightly undercounts predators by excluding some predators from trophically mixed orders such as Coleoptera, a previously published stable-isotope analysis of these same samples [17] showed that such miscategorizations represented a small fraction (∼5%) of all insects.

We sampled aerial arthropods (*N* = 3,277) from July 2007 to February 2008 using 10×13 cm yellow sticky traps (Olson Products, Medina, Ohio, USA). Each month (except August 2007), we hung one sticky trap at chest height at both 10 m and 30 m from the center of each of 12 mounds. Trap locations were random with respect to prevailing wind direction, and we marked the side of each trap that was facing towards the mound. We collected all traps after 24 h and identified and counted all arthropods. We analyzed log-transformed data using repeated-measures MANOVA (in JMP 8.01) with arthropod abundances in each month as the dependent variables. The between-subjects factors in this analysis were mound proximity, trap orientation (i.e., facing towards or away from mound), their interaction, and mound identity (because individual termite mounds vary in size and primary productivity). The within-subjects factor was time. The number of known predators (182 spiders) captured using this method was insufficient for separate analysis.

We sampled arboreal arthropods (*N* = 1,503) by spraying tree stems and canopies with 0.6% alphacypermethrin from a backpack sprayer [17]. Trees were selected randomly subject to the criteria that they were approximately 1–2 m tall (mean ± SD: 1.73±0.25 m) and occupied by the most common *Acacia-*ant symbiont, *Crematogaster mimosae*. Prior to spraying, we arranged white sheets beneath the canopies. On calm days, we sprayed each tree for 30 s and collected all arthropods falling onto the sheets during the subsequent 30 min. We sampled 10 trees at each of four mounds in July 2007 (a wet period) and an additional 10 trees at each of three mounds in February 2008 (a dry period), for a total of 70 trees at seven mounds. We measured the distance from each tree to the nearest mound center. After identifying and counting all samples, we dried them to constant mass at 60°C and weighed them (nearest 0.0001 g) to obtain separate dry-biomass measures for prey and predators. We constructed candidate sets of multiple regressions and selected the best models for subsequent analyses (see “Regression Modeling of Response Variables,” below). Because we sampled only similarly sized trees, there were no statistically significant pairwise correlations between tree size and either mound proximity or the arthropod response variables (all *p*≥0.25), although tree size did appear as a predictor in the best-fitting (as determined by AIC*_c_*) models of total-arthropod abundance and predatory-arthropod biomass (Table S3).

### Gecko Surveys

In 2006, as part of a concurrent study, Doak, Brody, and Palmer used a laser rangefinder (accurate to within 10 cm) to map and individually number all trees within ∼35-m-radius semicircles centered on each of six mounds. For this study, we selected three of these mounds and used a random-number generator to choose 60 trees (>1 m tall) for search. The mounds were several hundred meters apart. From July–August 2007, Pringle and two assistants exhaustively searched all trees for geckos, using ladders to reach high branches and probing within any hollows. For all 180 trees, we recorded the number of geckos, mound proximity (nearest 0.1 m), nearest-neighbor distance (nearest 0.1 m), height (nearest 0.1 m), basal diameter (nearest 0.1 cm), and resident *Acacia-*ant species. In August 2009, we repeated this process for an additional 477 trees at the same three termite mounds to obtain an independent dataset with which to test the predictive power of our best-fitting model of gecko abundance.

### Spider Fecundity

Female spiders guarding egg masses were selected opportunistically and haphazardly. Upon collection, we preserved female spiders in ethanol, placed the egg masses in ventilated plastic cups in a common laboratory environment, and checked them periodically. When we were confident that all spiderlings had emerged from the egg sacs (∼14 d after first emergence), we froze the spiderlings and counted them using a dissecting microscope. It is extremely unlikely that cannibalism among spiderlings during this interval influenced our results; we are not aware of any reports of cannibalism among newly hatched juveniles in the Araneidae, and a bias would require that cannibalism was much more frequent among offspring of females far from termite mounds, which is improbable. Of 110 egg cases, 106 (96%) hatched in the laboratory. We calculated two measures of reproductive output for each female: total number of spiderlings and mean number of spiderlings per egg sac per female (each female's egg mass consisted of 1–12 individual egg sacs).

Jocqué confirmed the genus identification for this as-yet-undescribed species and measured the width of the carapace and the combined length of the tibia and patella of leg I for each adult female. Measurements were made with an ocular graticule in a Leica M10 stereo microscope (measurement unit = 0.0164 mm). We could not obtain reliable carapace-width measurements for four females, giving us a final sample size of 102. Both measures of reproductive output were positively correlated with female carapace width (*r* = 0.24, *F*_1,100_ = 6.1, *p* = 0.02 and *r* = 0.20, *F*_1,100_ = 4.3, *p* = 0.04, respectively), while neither measure of reproductive output varied with tibia + patella length (both *p*≥0.5). Female carapace width was not significantly correlated with termite-mound proximity (*r* = −0.11, *F*_1,100_ = 1.3, *p* = 0.3).

### Regression Modeling of Response Variables

To determine the mechanisms (especially the role of termite-mound proximity) influencing tree-dwelling-arthropod abundance, gecko occurrence, and spider fecundity, we constructed sets of candidate regression models and ranked them using the AIC_c_. Prior to constructing candidate sets, we visually examined the shape of the relationship between each response variable and mound proximity. In all candidate sets, we included both a raw mound-proximity term and one-or-more nonlinear transformations (log_e_ for gecko abundance; square-root for spider abundance; log_e_, square, and square-root for arthropod abundance/biomass and *Cyclosa* fecundity; Tables S1–S3), as well as categorical mound-identity effects and (for all variables except spider fecundity) raw and transformed effects of tree size. Complete model sets and AIC_c_ results are available from Pringle on request.

To explain variation in the number of geckos on trees, we employed ordinal logistic regression using the “Ordinal” routine in the Statbox 4.2 Toolbox for MATLAB (http://www.statsci.org/matlab/statbox.html). The dependent variable—number of geckos per tree in our dataset of 180 trees at three mounds—took values 0, 1, 2, or≥3. Independent variables included combinations of mound proximity, tree size (i.e., estimated surface area of the main stem, using the equation for the area of the side of a cylinder, which we considered a more accurate representation of gecko habitat size than either height or diameter alone), distance to the nearest tree ≥1 m tall, and mound identity. We constructed 108 candidate models using combinations of these variables, their natural logarithms, and their first-order interactions. We then ranked these models using AIC_c_ (Table S1). Our notation and interpretation follow Burnham and Anderson [29]. Of the five most likely models, all contained terms for both tree size and mound proximity (Table S1). Examination of the complete model set revealed that the importance of variables decreased in the order: tree size > mound proximity > mound identity > nearest-tree distance. We evaluated the goodness-of-fit and predictive ability of our best model by comparing mean model predictions with mean observed results for 12 different categories of trees (assigned based on which of three mounds and which of four 10 m distance intervals they belonged to). We performed this test using both the original 180-tree dataset from 2006 (which reveals goodness-of-fit, Figure S3A) and a novel 477-tree dataset from 2009 (which reveals the substantial predictive power of our model: Figure S3B).

We conducted multiple-regression analyses of the abundance of adult arboreal spiders (based on our sample of 70 trees that we sprayed with insecticide) that largely paralleled our ordinal-regression analyses of gecko abundance. Independent variables included combinations of mound proximity, estimated tree surface area, square-root transformations of these variables, their first-order interactions, and mound identity. We constructed 26 candidate models and ranked them using AIC_c_ (Table S2). Of the eight most likely models, all contained terms for mound proximity and mound identity (which encompassed seasonal variations in abundance); no model lacking a term for mound proximity received any empirical support. Examination of the complete model set revealed that variable importance decreased in the order: mound identity ≈ mound proximity > tree size.

We analyzed arthropod abundance and biomass data (log-transformed to meet parametric assumptions) using multiple regression. Response variables included total arthropod abundance and biomass, prey-arthropod abundance and biomass, and predatory-arthropod abundance and biomass. We constructed 24 candidate models for each variable. Unlike for geckos and spiders alone, all models for arthropod abundance/biomass contained a mound-proximity term (either raw or transformed, as described above), but none contained interactions. The other predictors included raw and log-transformed tree size (estimated surface area, as described above) and mound identity. The best models (Table S3) explained between 2% (for prey-arthropod biomass) and 68% (for predatory-arthropod abundance) of the variation in the response variables. For spider fecundity, we constructed 16 candidate models using raw and transformed mound proximity, female carapace width, and mound identity as predictors. The best model (Table S3) explained 23% of the variation in spider fecundity.

For each response variable, we used the single best model for all spatial analyses (see below) and all tests of statistical significance for individual predictors. The log-transformed mound-proximity term was a better predictor of gecko abundance than the linear form. Square-transformed mound-proximity terms best approximated the responses of all arthropod variables except predator abundance, which was best approximated by a linear term, and prey biomass, which was best approximated (albeit non-significantly) by a log-transformed term.

### Spatial Analysis of Patterns in Consumer Abundance

We extrapolated to the landscape scale for six response variables (predatory-arthropod abundance/biomass, total arthropod abundance/biomass, gecko occurrence, and spider fecundity) using a 600×600 m section of our study area, which included 62 termite mounds (Figure 1A). We mapped this area using Quickbird satellite imagery and calibrated the map in the field with a laser rangefinder. We subdivided this area with a grid of 5×5 m cells that defined 14,400 sample points and computed the distance of each point to the nearest mound. We then applied the best-fitting regression model to the distance value for each point. This enabled us to produce the spatial probability distribution of gecko abundance in Figure 2B and also to compute the mean value across all points for each response variable. In making these estimates, we used the observed median value of all other predictor variables (which, depending on the model in question, included tree size, spider-carapace width, and categorical mound-identity effects: Tables S1–S3). In other words, although we refer to these estimates as “real-” or “over-dispersed-landscape” values, they actually estimate abundances in hypothetical landscapes in which the distribution of mounds corresponds to reality, but all trees, female spiders, etc. are assumed to be an identical, typical size, and tree density is assumed to be uniform throughout the landscape (we provide support for this last and most-important assumption below).

We then compared the estimated mean landscape value of each response variable from the actual, over-dispersed mound landscape with the corresponding distributions of values from simulated random landscapes that lacked the uniform spacing of real mounds (Figure 4). To do this, we generated 1,000 hypothetical landscapes that had the same number of mounds (*N* = 62) as the real landscape, but a nearly Poisson (independent and random) distribution of the mounds. To generate these landscapes, we randomly picked sets of latitudes and longitudes to define the location of each mound center within the same size area as that actually surveyed. The only restriction on these randomly generated mound positions was that all mound centers be at least 10 m apart, since real mounds have radii of 5–10 m and cannot overlap. For each simulated mound landscape, we repeated the estimation procedure (described above for the actual landscape) to produce 1,000 hypothetical landscape-wide average values for each response variable. Comparing the mean estimated values of all sample points from the 1,000 hypothetical random landscapes with the mean values obtained from the actual, over-dispersed landscape generated the results shown in Figure 4.

As mentioned above, the most likely real-world complication that could influence the results of the randomization tests just described is variation in tree densities at different distances from mounds. For one mound in our study area, we mapped the positions of all trees out to ∼35 m in all directions; for five other mounds, we did the same for a ∼35 m radius semicircle. We used these data to determine whether and how the densities of trees >1 m tall vary with distance from mound centers. We used the following procedure to determine densities. First, for each mound, we used a MATLAB routine to construct Voronoi or Thiessen polygons [41] around each tree in the mapped area, which provides an estimate of local, tree-specific density. Next, we constructed a convex-hull line between the trees that defined the outermost boundaries of the mapped area. Because Voronoi polygons cannot be accurately estimated around these boundary trees, we eliminated these trees from further analyses. We then binned the remaining trees into either 5 or 10 m distance bins and divided the number of trees in each bin by their summed polygon areas to arrive at a distance-specific tree-density estimate.

Using these estimates, we applied general linear models with distance and mound identity as independent variables and density as the dependent variable. For 5 m bins, mound ID is highly significant (*p*<10^−7^) and distance is also significant (*p* = 0.01), with density increasing on average with distance, but with much variation between mounds (no significant interaction effect). For 10 m bins, mound ID, distance, and their interaction are significant (mound: *p*<10^−7^; distance: *p* = 0.03; interaction: *p* = 0.00003). For these results, removal of the interaction effects makes the main effect of distance non-significant due to strongly varying patterns in density across mounds.

These highly variable results make it unlikely that any consistent patterns in tree density would bias the conclusions of our simulations. However, to test for such effects, we used the results from the 5 m binned data to estimate changes in densities for the average mound effect (density = 0.071+0.0011×distance). We used this relationship to estimate a weighted average of all sampled points in the real and simulated landscapes that accounted for the relative tree density at different distances. These results (Figure S5) are qualitatively identical to our original results (Figure 4).

### Gecko Habitat-Manipulation Experiments

Our experiments were designed to (a) isolate the effects of tree size and mound proximity on gecko occupation rates and (b) determine whether mound proximity truly represented a trophic effect. We created artificial gecko habitat using wooden posts of two different sizes. “Large” posts were 2.6±0.06 m tall and 10.3±0.58 cm in diameter (means ± SD) (Figure S1B). “Small” posts were 2.0±0.03 m tall and 7.7±0.35 cm in diameter (Figure S1D). All posts contained 12 1.5 cm diameter holes for refuge and a 1 m long horizontal crossbar to provide a perch. These posts were very similar to trees from the geckos' perspective, as we determined after the experiment by comparing occupancy of the 48 posts (over the first 12 surveys) with 48 real trees that matched in size (mean estimated surface area = 0.67 m^2^ for both real and artificial trees; mean occupancy = 0.6±0.2 and 0.7±0.1 geckos/tree, respectively, means±95% CI). At each of 12 termite mounds, we placed one post of each size at both 10 m (“close”) and 30 m (“far”) from the mound center. We placed the large and small posts 5 m from one another at each distance. To control for any confounding influence of neighboring tree density, we situated each post 3 m from the nearest tree ≥2 m tall and ensured that the density of trees in the 20×20 m area surrounding the posts at each distance did not differ (close density = 23.8±6.6, far density = 24.2±6.0, means ± SD).

We completed the experimental setup on September 30, 2006 and waited 1 mo prior to beginning surveys to allow geckos to adjust to the habitat perturbation and colonize the posts. Between October 28, 2006 and June 20, 2007, we conducted 12 surveys of all posts. Because of the simplified architecture of the posts, we suspect that detection probability approached 100%. During five of these surveys, we captured geckos (*N* = 134), which we sexed, measured (nearest 1 mm), weighed (nearest 0.001 g), and replaced. To avoid pseudoreplication arising from multiple counts of the same individuals, we treated the posts as the experimental units: our response variables were mean adult gecko length and weight (by sex) and mean occupation frequency (number of geckos observed on each post divided by 12, the number of surveys) of each post. (Because up to three geckos sometimes occurred simultaneously on a single post, occupation frequency could take values >1.) Size and weight data were compared using two-way factorial ANOVA.

To ascertain whether the effect of mound proximity on gecko occupation arose from variation in prey availability, we repeated this experiment in conjunction with daily food supplementation. Insects, which included mealworms, termite workers found in dried dung, and sweep-net contents (all collected off site), were always added to the cups between 7:30 and 8:30 a.m., immediately prior to the onset of peak gecko activity. We did not attempt to capture any geckos during this phase of the experiment (Text S3). As before, we conducted 12 surveys of all posts. Mean monthly rainfall did not differ between the pre- and post-prey-addition periods (63.2±46.1 mm and 52.4±34.9 mm, respectively; *F*_1,12_ = 0.2, *p* = 0.7).

We analyzed the data from both runs of this experiment using a single repeated-measures MANOVA design (in JMP 8.01). The dependent variables were the mean occupation frequencies of each post during the 12 surveys prior to prey addition and the same mean frequencies for the 12 surveys conducted during daily prey addition to the far posts. The between-subject factors were post size (large versus small), mound proximity (close versus far), their interaction, and mound identity. The within-subject factor was time (pre- versus post-prey addition). In this design, the significant time × mound proximity interaction (Table S3) shows the equalizing effect of experimental food supplementation.

## Supporting Information

Figure S1 **Contextual photographs.** (A) Aerial view of apparently homogeneous black-cotton ecosystem. (B) Ground view of *Odonotermes* mound (white arrow pointing to dark-green vegetation patch) and "large-close" experimental post (foreground). (C) Portion of excavated termite mound showing fungus-comb chambers (white arrows). (D) *Lygodactylus keniensis* gecko occupying a "small" experimental post.
(6.06 MB TIF)

Figure S2 **Results of Ripley's** ***K*** **-function analysis of termite mounds in a ∼3-km^2^ portion of the landscape that includes our study area.** *L*(d) values (a transformation of Ripley's *K* for which zero indicates the number of neighbors expected in a random landscape, negative values indicate fewer-than-expected neighbors, and positive values indicate more neighbors than expected) are plotted against distance. Dashed red lines represent 95% confidence limits expected from a random landscape, solid black line represents observed *L*(d). The significantly lower-than-expected values of *L* at scales < 100 m indicate even spacing, while the significantly higher-than-expected values at scales >300 m reflect clustering at the landscape scale. Thus, evenly spaced lattices of mounds are embedded in a landscape in which overall mound density varies (perhaps as a function of resource availability). Note that the minimum value of *L* (reflecting maximally even spacing) occurs at a spatial scale of approximately 30 m, which corresponds to the mean distance to the nearest mound center in the mapped portion of the landscape (29.22 m).
(0.17 MB TIF)

Figure S3 **Goodness of fit and predictive power of gecko model.** We binned all trees into 12 categories based on which of three mounds (M3, M6, and M19) and four distance categories (0-10 m, 10-20 m, 20-30 m, and 30-40 m) they belonged to. For each of these categories, we calculated the observed mean number of geckos per tree and plotted the values against those predicted by the model. A 1:1 line, indicating perfect correspondence between model predictions and results, is plotted for comparison. (A) Goodness-of-fit. Based on the original 2006 data from 180 trees at three mounds, which was used to parameterize the model (correlation coefficient: *r*  =  0.65; *r*  =  0.91 when the major outlier, a category with only 5 trees, is excluded). (B) Predictive power. We applied the same model (with identical parameters) to a novel dataset of 477 trees at the same three mounds, collected in August 2009 (correlation coefficient: *r*  =  0.75).
(0.18 MB TIF)

Figure S4 **Dependence of response variables on mean distance to nearest mound.** Dependence of mean values of response variables on mean distance to nearest mound in 1,000 simulated random landscapes for (A) total-arthropod biomass, (B) predatory-arthropod biomass, (C) gecko abundance, and (D) spider fecundity. For each artificial landscape, generated by the random placement of mound locations, the mean distance to the nearest mound (horizontal axis) and the landscape-wide mean of the response variable (vertical axis) are plotted. The distribution of points in (A), (B), and (D) is identical due to the shared form of the best-fitting multiple-regression model for these variables. The scatterplots for total- and predatory-arthropod abundance (not shown) are similar to those for biomass in (A-B). These results show that average measures of community productivity are greatest in simulated landscapes in which mounds were by chance more over-dispersed, and that landscape-scale productivity decreases with increasing aggregation of mounds, because clumping results in greater average distance to the nearest mound center.
(0.22 MB TIF)

Figure S5 **Tree-density corrected simulation results.** Frequency distributions of mean landscape values in 1,000 simulated landscapes of randomly placed mounds in which we controlled for variation in tree density with distance from termite mounds (cf. Fig. 4 and Materials & Methods: Spatial analysis of patterns in consumer abundance). (A) Total-arthropod biomass, (B) predatory-arthropod biomass, (C) geckos, (D) spider fecundity, (D) predatory-arthropod abundance, (E) total-arthropod abundance. Vertical bars show the mean landscape values for each variable obtained using the evenly spaced distribution of termite mounds in the mapped 0.36-km^2^ area of the landscape (Figure 1A). The best-fitting models used in the analyses are presented in Tables S1-S3.
(0.79 MB TIF)

Table S1 **Top five ordinal-regression models of gecko abundance.** The five ordinal regression models of gecko occupancy patterns that received "substantial" empirical support (*Δ_i_* < 2) according to the second order Akaike Information Criterion (AIC_c_), along with a model (for comparison) that contained only a constant. The best-fitting model, which we used for simulations, appears in bold. *Δ_i_* is the difference between a model's AIC_c_ value and that of the model with the lowest AIC_c_; the Akaike weight *w_i_* is likelihood of a given model's being the best model in the set [ref. 29, main text]. Examination of the full set of 108 candidate models shows that Tree Size and Mound Proximity (in that order) were by far the most important predictors of gecko abundance.
(0.02 MB XLS)

Table S2 **Top eight ordinal-regression models of spider abundance.** The eight ordinal regression models of adult spider occupancy that received "substantial" empirical support (*Δ_i_* < 2) according to the second order Akaike Information Criterion (AIC_c_), along with a model (for comparison) that contained only a constant. The best-fitting model, which we used for simulations, appears in bold. Symbols correspond to those in Table S1. Examination of the full set of 26 candidate models shows that Mound Proximity and Mound Identity are the most important predictors of spider abundance; the best-fitting model that did not include a term for distance (which contained the predictors Mound Identity and square-root-transformed Tree Size) had *Δ_i_*  = 32.92 and *w_i_*  =  0.000, indicating essentially zero empirical support [ref. 29, main text].
(0.02 MB XLS)

Table S3 **Best-fitting multiple-regression models for arthropod response variables.** Best-fitting multiple-regression models for arthropod response variables, which we used in simulations for each variable, with P values from effect tests on the term for mound proximity. Best-fitting models were selected from candidate sets of 24 (for abundance/biomass measures) or 16 (for spider fecundity) using AIC_c_, as described in the Materials and Methods. The model for prey-arthropod biomass appears in italics because it explains a trivial amount of the variance, and because the negative relationship with mound proximity was not statistically significant.
(0.02 MB XLS)

Table S4 **Repeated-measures MANOVA table for gecko occupancy in the habitat-selection experiment.** The dependent variables were (a) the mean occupation frequencies for each post during the 12 surveys prior to prey addition and (b) the same frequencies for the 12 surveys conducted during daily prey addition to far posts only. The Time*Proximity interaction term is significant because the experimental addition of prey equalized gecko occupancy rates at 10 m and 30 m from termite mounds (Fig. 3A, main text).
(0.02 MB XLS)

Text S1 **Mechanism underlying patterning of termite mounds.**
(0.05 MB DOC)

Text S2 **Potential direct effects between termites and predators.**
(0.03 MB DOC)

Text S3 **Interpretation of gecko habitat-selection experiment.**
(0.02 MB DOC)
