# Supplementary figures and images for: Spatial Pattern Enhances Ecosystem Functioning in an African Savanna
Source: PLoS Biol. 2010 May 25;8(5):e1000377. doi: 10.1371/journal.pbio.1000377 (PMC2876046; doi:10.1371/journal.pbio.1000377)

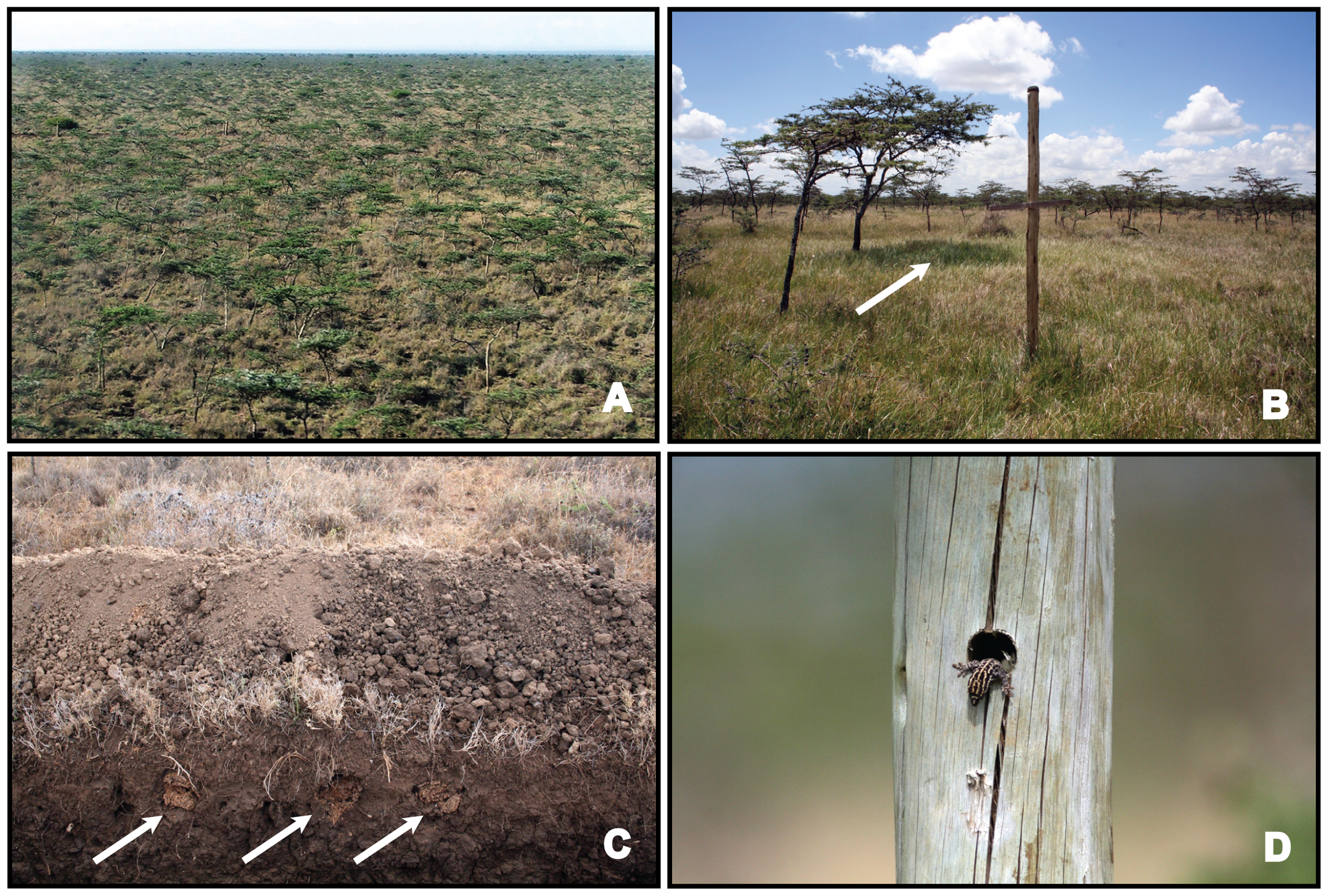

Supplement: Figure S1 — Contextual photographs. (A) Aerial view of apparently homogeneous black-cotton ecosystem. (B) Ground view of Odonotermes mound (white arrow pointing to dark-green vegetation patch) and "large-close" experimental post (foreground). (C) Portion of excavated termite mound showing fungus-comb chambers (white arrows). (D) Lygodactylus keniensis gecko occupying a "small" experimental post. (6.06 MB TIF) [file pbio.1000377.s001.tif]

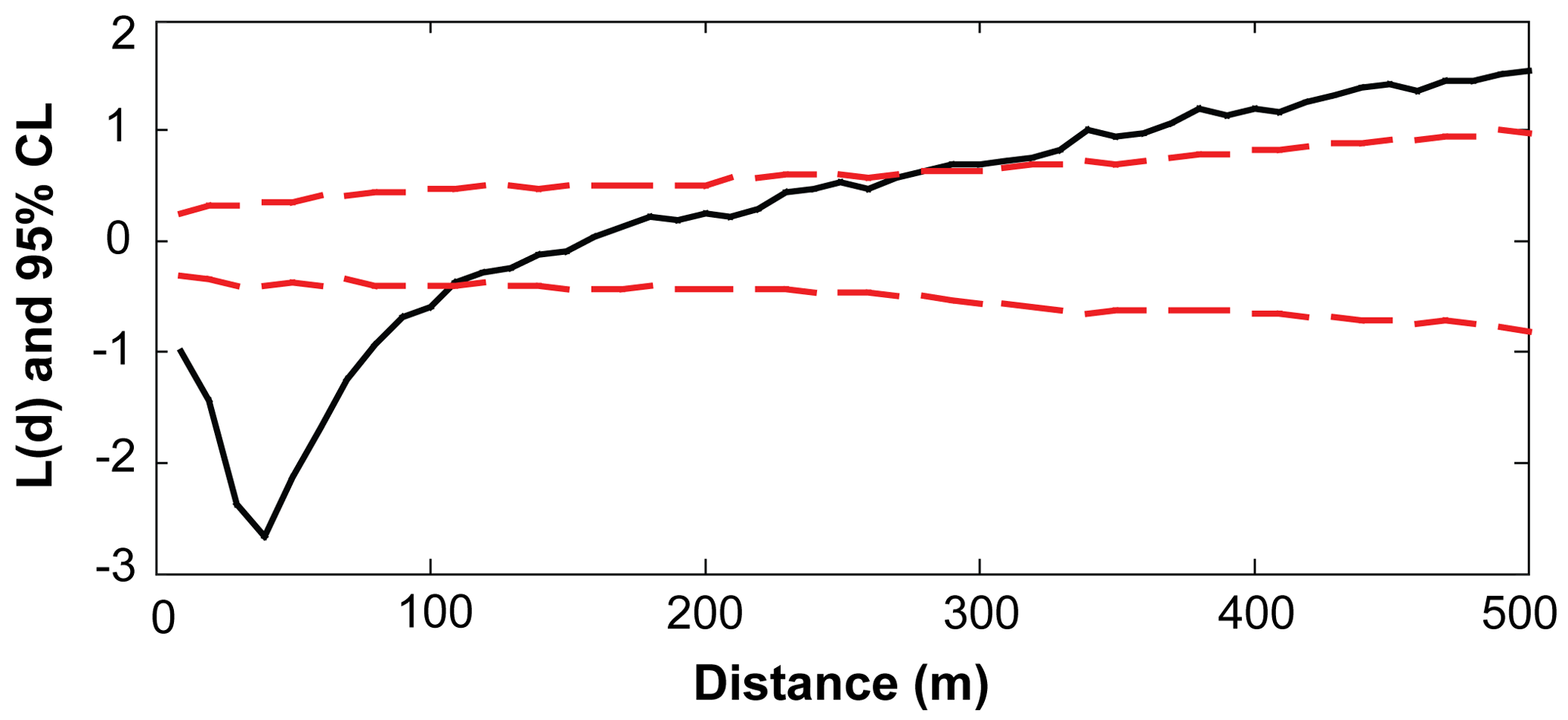

Supplement: Figure S2 — Results of Ripley's K -function analysis of termite mounds in a ∼3-km2 portion of the landscape that includes our study area. L(d) values (a transformation of Ripley's K for which zero indicates the number of neighbors expected in a random landscape, negative values indicate fewer-than-expected neighbors, and positive values indicate more neighbors than expected) are plotted against distance. Dashed red lines represent 95% confidence limits expected from a random landscape, solid black line represents observed L(d). The significantly lower-than-expected values of L at scales < 100 m indicate even spacing, while the significantly higher-than-expected values at scales >300 m reflect clustering at the landscape scale. Thus, evenly spaced lattices of mounds are embedded in a landscape in which overall mound density varies (perhaps as a function of resource availability). Note that the minimum value of L (reflecting maximally even spacing) occurs at a spatial scale of approximately 30 m, which corresponds to the mean distance to the nearest mound center in the mapped portion of the landscape (29.22 m). (0.17 MB TIF) [file pbio.1000377.s002.tif]

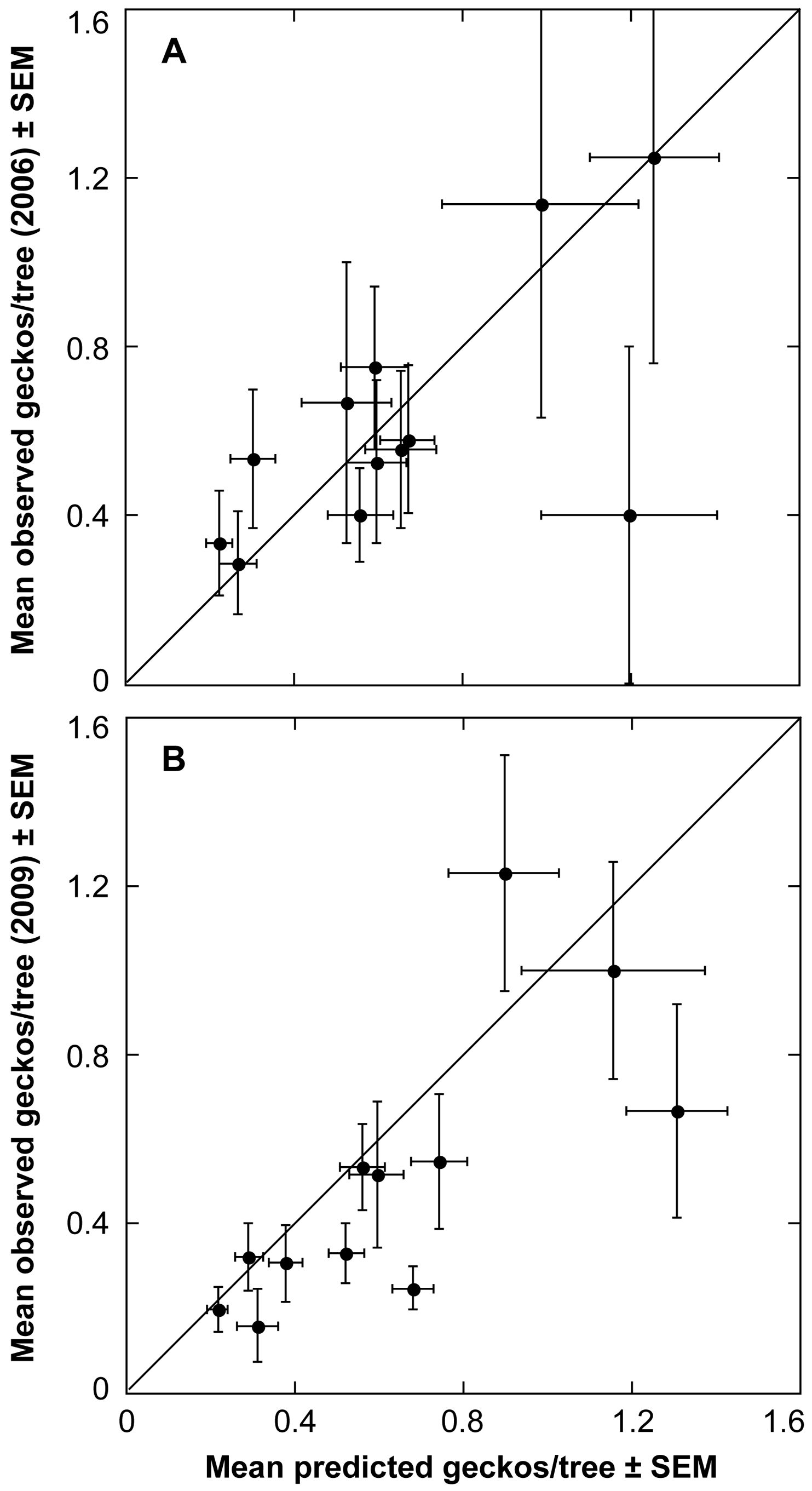

Supplement: Figure S3 — Goodness of fit and predictive power of gecko model. We binned all trees into 12 categories based on which of three mounds (M3, M6, and M19) and four distance categories (0-10 m, 10-20 m, 20-30 m, and 30-40 m) they belonged to. For each of these categories, we calculated the observed mean number of geckos per tree and plotted the values against those predicted by the model. A 1:1 line, indicating perfect correspondence between model predictions and results, is plotted for comparison. (A) Goodness-of-fit. Based on the original 2006 data from 180 trees at three mounds, which was used to parameterize the model (correlation coefficient: r = 0.65; r = 0.91 when the major outlier, a category with only 5 trees, is excluded). (B) Predictive power. We applied the same model (with identical parameters) to a novel dataset of 477 trees at the same three mounds, collected in August 2009 (correlation coefficient: r = 0.75). (0.18 MB TIF) [file pbio.1000377.s003.tif]

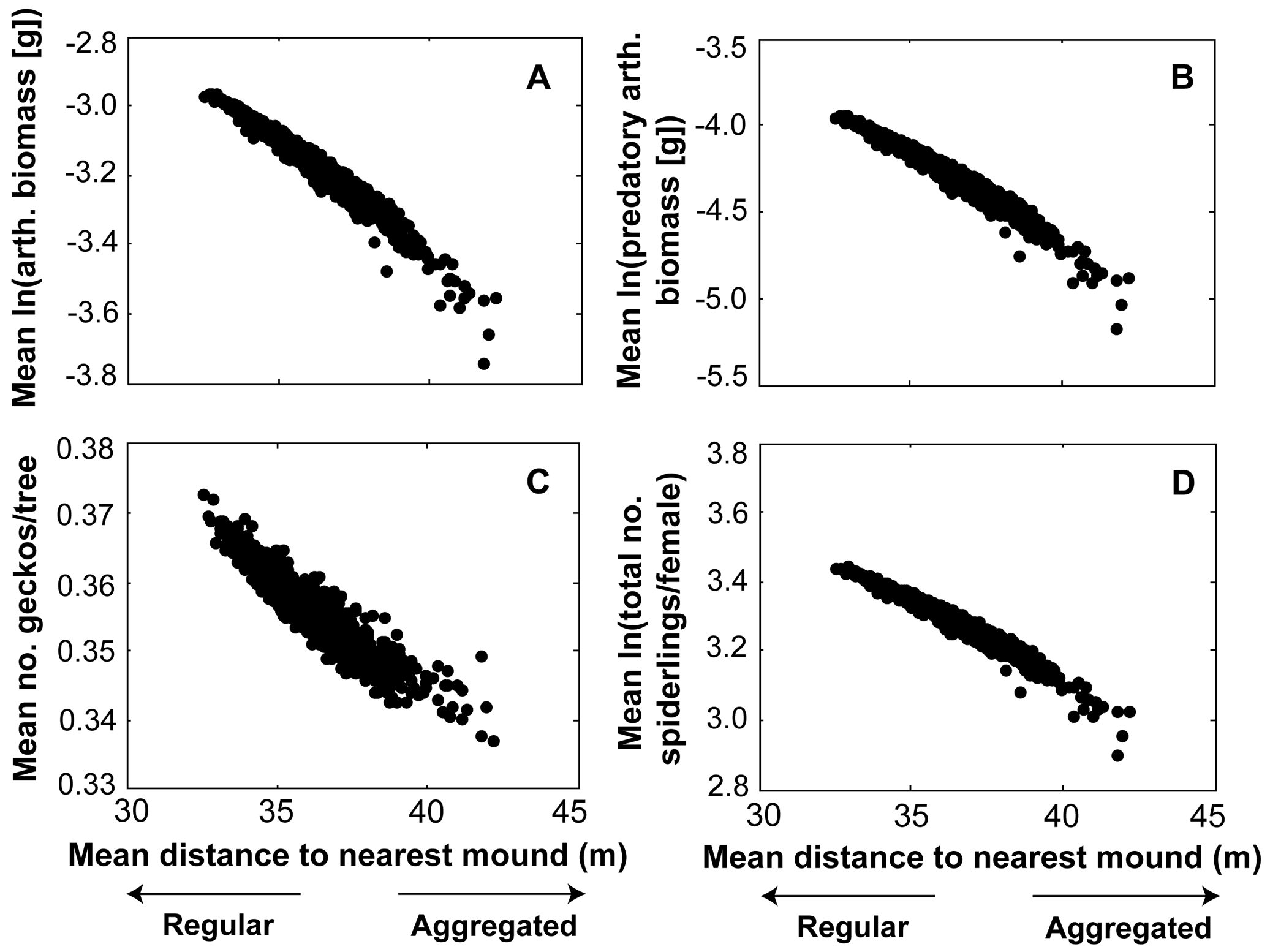

Supplement: Figure S4 — Dependence of response variables on mean distance to nearest mound. Dependence of mean values of response variables on mean distance to nearest mound in 1,000 simulated random landscapes for (A) total-arthropod biomass, (B) predatory-arthropod biomass, (C) gecko abundance, and (D) spider fecundity. For each artificial landscape, generated by the random placement of mound locations, the mean distance to the nearest mound (horizontal axis) and the landscape-wide mean of the response variable (vertical axis) are plotted. The distribution of points in (A), (B), and (D) is identical due to the shared form of the best-fitting multiple-regression model for these variables. The scatterplots for total- and predatory-arthropod abundance (not shown) are similar to those for biomass in (A-B). These results show that average measures of community productivity are greatest in simulated landscapes in which mounds were by chance more over-dispersed, and that landscape-scale productivity decreases with increasing aggregation of mounds, because clumping results in greater average distance to the nearest mound center. (0.22 MB TIF) [file pbio.1000377.s004.tif]

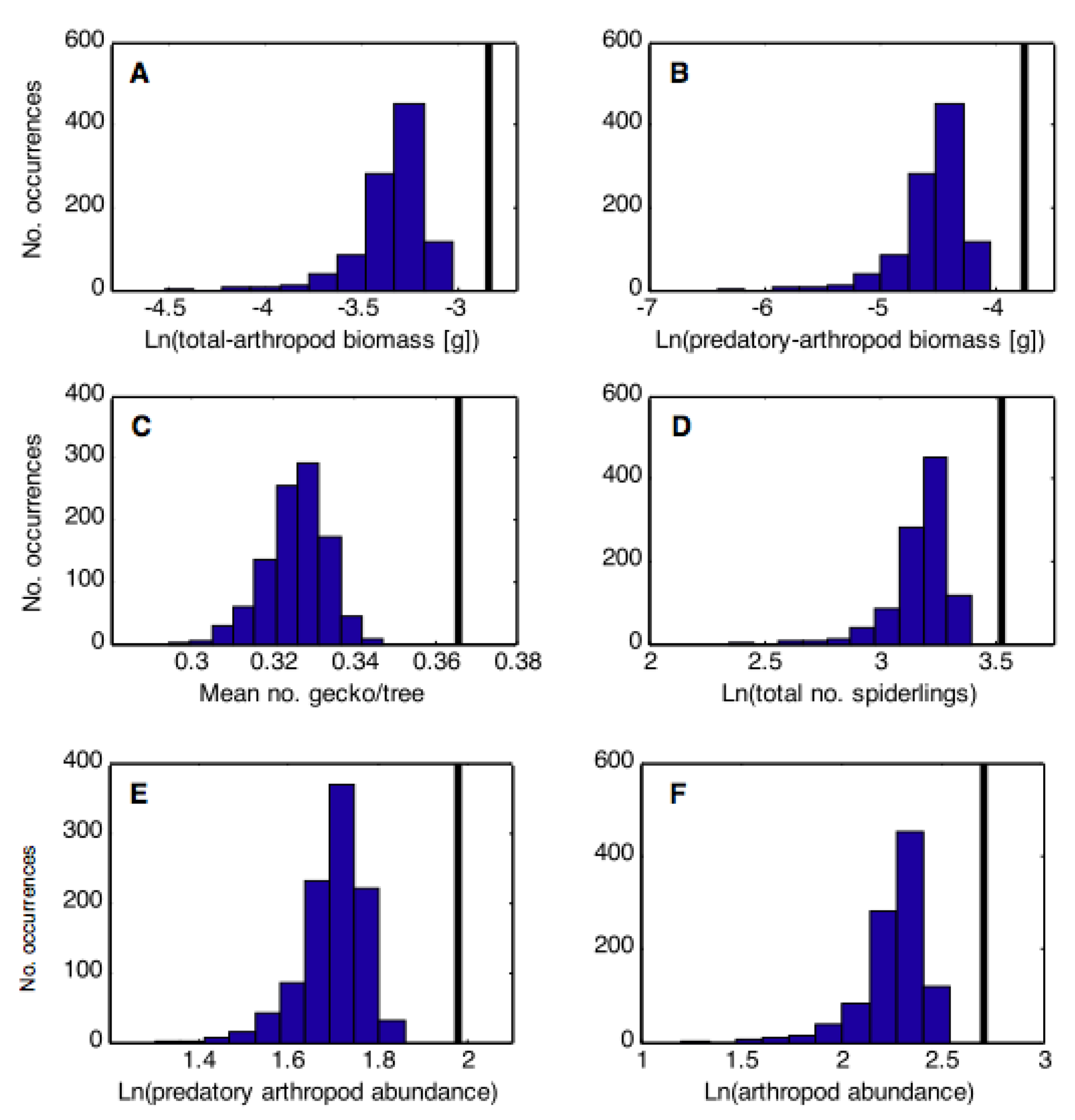

Supplement: Figure S5 — Tree-density corrected simulation results. Frequency distributions of mean landscape values in 1,000 simulated landscapes of randomly placed mounds in which we controlled for variation in tree density with distance from termite mounds (cf. Fig. 4 and Materials & Methods: Spatial analysis of patterns in consumer abundance). (A) Total-arthropod biomass, (B) predatory-arthropod biomass, (C) geckos, (D) spider fecundity, (D) predatory-arthropod abundance, (E) total-arthropod abundance. Vertical bars show the mean landscape values for each variable obtained using the evenly spaced distribution of termite mounds in the mapped 0.36-km2 area of the landscape (Figure 1A). The best-fitting models used in the analyses are presented in Tables S1-S3. (0.79 MB TIF) [file pbio.1000377.s005.tif]
