# Supplementary material for: Spatial Pattern Enhances Ecosystem Functioning in an African Savanna
Source: PLoS Biol. 2010 May 25;8(5):e1000377. doi: 10.1371/journal.pbio.1000377 (PMC2876046; doi:10.1371/journal.pbio.1000377)
Supplement: Text S1 — Mechanism underlying patterning of termite mounds. (0.05 MB DOC) [file pbio.1000377.s010.doc]

**Text S1**

**Mechanism underlying patterning of termite mounds.** The origin of spatial pattern in termite mounds does not affect the validity of our results or interpretations—our conclusions about the emergent effects of spatial pattern should hold whether the patterns are generated endogenously, exogenously, or anthropogenically. Nevertheless, because the question invariably arises, we briefly review the evidence that mound spatial pattern is endogenous and self-organized.

For the pattern of termite mounds and their associated plant and animal communities to fit the definition of self-organization, it must arise from local species interactions rather than a pre-existing geophysical template [1]. The “regular polka-dot pattern” [2] of termite mounds is a well-known phenomenon [3-6], and a substantial literature documents—both directly via experimentation and indirectly via observation and inference—the role of exploitation and/or interference competition in producing such spatial patterns, in both African Macrotermitinae [2,7-11] and other termite taxa worldwide [12-14]. The one study, to our knowledge, that questions the role of competition in determining mound spacing [15] was based on an observation of mounds that were not evenly spaced, a finding that has itself been questioned [9]. In any event, the mounds at our site are clearly evenly spaced at spatial scales < 100 m (Figure S2 and Figure 1A in main text).

We lack a plausible alternative geophysical explanation for the establishment and maintenance of this uniform pattern. The soil at our site is strikingly homogeneous and lacks macrotopography of any kind. Although soil composition is somewhat variable at the landscape scale [16], there is no indication that this variability is patterned, regularly or otherwise. Some high-clay vertisols, via shrink-swell dynamics, generate microtopographic bumps and depressions called gilgai [17]. These features may in some cases form regular patterns, but they are not coincident with *Odontotermes* mounds in other parts of Kenya [3], nor can we think of any reason why they would be. Furthermore, the distribution of gilgai should occur on a smaller spatial scale than that of termite mounds and would generate vegetation patterns in opposition to the patterns that we observe with termite mounds [3]. Moreover, even spacing of termite mounds in upland Kenya is evident in sites with clay concentrations too low to generate gilgai [3,5]. Thus, the most parsimonious explanation for the over-dispersion of *Odontotermes* mounds at this site is as the endogenous product of competitive interactions among termite colonies.

**Text S1 References**

1. Rietkerk M, van de Koppel J (2008) Regular pattern formation in real ecosystems. Trends Ecol Evol 23: 169-175.

2. Jones JA (1990) Termites, soil fertility and carbon cycling in dry tropical Africa: a hypothesis. J Trop Ecol 6: 291-305.

3. Darlington JPEC (2005) Termite nest structure and impact on the soil at the radar site, Embakasi, Kenya (Isoptera: Termitidae). Sociobiology 45: 521-542.

4. Glover PE, Trump EC, Wateridge LED (1964) Termitaria and vegetation patterns on the Loita Plains of Kenya. J Ecol 52: 367-377.

5. Darlington JPEC (1985) Lenticular soil mounds in the Kenya highlands. Oecologia 66: 116-121.

6. McFarlane MJ, Darlington JPEC (1989) An example of termite mounds as indicators of hydrogeological and soil conditions in Malawi and Zimbabwe. Sociobiology 15: 271-272.

7. Darlington JPEC (1982) The underground passages and storage pits used in foraging by a nest of the termite *Macrotermes* *michaelseni* in Kajiado, Kenya. J Zool 198: 237-247.

8. Korb J, Linsenmair KE (2001) Resource availability and distribution patterns, indicators of competition between *Macrotermes bellicosus* and other macro-detritivores in the Comoe National Park, Cote d'Ivoire. Afr J Ecol 39: 257-265.

9. Korb J, Linsenmair KE (2001) The causes of spatial patterning of mounds of a fungus-cultivating termite: results from nearest-neighbour analysis and ecological studies. Oecologia 127: 324-333.

10. Pomeroy DE (2005) Dispersion and activity patterns of three populations of large termite mounds in Kenya. J East Afr Nat Hist 94: 319-341.

11. Bloesch U (2008) Thicket clumps: A characteristic feature of the Kagera savanna landscape, East Africa. J Veg Sci 19: 31-44.

12. Levings SC, Adams ES (1984) Intraspecific and interspecific territoriality in *Nasutitermes* (Isoptera, Termitidae) in a Panamanian mangrove forest. J Anim Ecol 53: 705-714.

13. Jones SC, Trosset MW (1991) Interference competition in desert subterranean termites. Entomologia Experimentalis Et Applicata 61: 83-90.

14. Leponce M, Roisin Y, Pasteels JM (1996) Intraspecific interactions in a community of arboreal nesting termites (Isoptera: Termitidae). J Insect Behav 9: 799-817.

15. Schuurman G, Dangerfield JM (1997) Dispersion and abundance of *Macrotermes michaelseni* colonies: a limited role for intraspecific competition. J Trop Ecol 13: 39-49.

16. Riginos C, Grace JB (2008) Savanna tree density, herbivores, and the herbaceous community: bottom-up vs. top-down effects. Ecology 89: 2228-2238.

17. Brady NC, Weil RR (2002) The nature and properties of soil. Old Tappan, New Jersey: Prentice Hall.
