# Supplementary material for: Spatial Pattern Enhances Ecosystem Functioning in an African Savanna
Source: PLoS Biol. 2010 May 25;8(5):e1000377. doi: 10.1371/journal.pbio.1000377 (PMC2876046; doi:10.1371/journal.pbio.1000377)
Supplement: Text S2 — Potential direct effects between termites and predators. (0.03 MB DOC) [file pbio.1000377.s011.doc]

**Text S2**

**Potential direct effects between termites and predators.** In the main text, we document the absence of termite workers/soldiers from arboreal microhabitats and conclude that the influence of termites on the distribution of aboveground consumers is indirect. This is certainly the case for non-predatory arthropods, but might be uncertain for predators if they ate large numbers of termites. The arboreal predators in our study do occasionally consume winged reproductive termites; we have observed them in spider webs following the first rains of the season. However, we do not consider predation upon alates a plausible mechanism for the patterning we describe, for two reasons. First, emergence events may be as rare as once or twice per year [1], yet the patterns we describe are present in all months. It is difficult to imagine that such rare predator-satiating events are sufficient to maintain year-round pattern among mobile insectivores that feed daily, such as geckos. Second, our earlier isotopic food-web reconstruction (based on data from both wet and dry seasons) showed that arboreal gecko, spider, and mantid diets consist ~90% of insects that feed almost exclusively on C_3_ plants [2]. In contrast, termites in this system derive ~60% of their diets from C_4_ grasses (D. Doak and K. Fox-Dobbs, unpublished isotopic data). Thus, termites are not appreciable diet sources for these predators, and we conclude that direct predation upon termites plays little, if any, role in the observed patterns of predator and prey abundance. (And even if termites *were* an appreciable food source for these predators, our most important result—that the uniform spacing of mounds increases landscape-level production—would remain unchanged).

**Text S2 References**

1. Darlington JPEC (1985) Lenticular soil mounds in the Kenya highlands. Oecologia 66: 116-121.

2. Pringle RM, Fox-Dobbs K (2008) Coupling of canopy and understory food webs by ground-dwelling predators. Ecol Lett 11: 1328-1337.
