# Supplementary material for: Spatial Pattern Enhances Ecosystem Functioning in an African Savanna
Source: PLoS Biol. 2010 May 25;8(5):e1000377. doi: 10.1371/journal.pbio.1000377 (PMC2876046; doi:10.1371/journal.pbio.1000377)
Supplement: Text S3 — Interpretation of gecko habitat-selection experiment. (0.02 MB DOC) [file pbio.1000377.s012.doc]

**Text S3**

**Interpretation of the repeated-measures gecko habitat-selection experiment.** The analysis of our repeated-measures experiment would be problematic if overall occupation frequencies had increased with time, due either to seasonal fluctuations in gecko abundance/activity or to geckos’ becoming progressively more willing to occupy experimental posts in lieu of natural trees (a “maturation effect”). In that case, we might not have been able to conclude that the addition of prey at 30 m *caused* the increase in occupation rates that we observed at “far” posts between the first and second phases of the experiment. In fact, however, mean occupation rates across all “close” posts (which received no supplemental prey) actually decreased slightly (from 0.83 to 0.80 geckos/post) from the first phase to the second. Indeed, geckos readily occupied the experimental posts from the outset of the experiment: mean occupancy of the 48 posts in the very first survey (28 October 2006) was identical (0.6 geckos/tree) to that of 48 real trees with the same mean size (0.67 m^2^). Thus, we rule out seasonal and maturation effects and conclude that the effects we observed were due to the daily experimental addition of prey.

The only other difference in protocol between the first and second phases of the experiment was that we did not attempt to capture and weigh geckos during the second (prey-addition) phase. However, if catching geckos during the first phase had decreased their occupation of the experimental posts, then we would again have expected across-the-board increases in occupation frequency during the prey-addition phase. As stated above, however, occupation frequency increased only at far posts (which received supplemental prey).

The only experimental results that we did not predict on the basis of our surveys and regression models were the significant interactions between post size and mound proximity and between time and post size (Table S2). We can only speculate about the reasons for these results. The significant proximity*size interaction arose because the difference in occupation frequency between large and small posts was more pronounced at 10 m than at 30 m from termite mounds. This might be because the large-close posts constituted exceptionally good territories and were almost always occupied, while small posts (in the absence of supplemental prey) represented relatively poor territory regardless of their proximity to mounds. The significant time*size interaction arose because occupation frequencies of small posts were greater at 30 m than at 10 m during the second (prey-addition) phase, while occupation rates of large posts remained greater at 10 m than at 30 m (although less dramatically so than in the first phase). This result might indicate that the repeated addition of prey to small posts provided a reliable resource that mitigated the disadvantages conferred by small territory size, whereas even repeated prey addition could not enhance the quality of large-far posts sufficiently to match the apparently ideal large-close posts. Further work is required to explain these interactions.
